# Comparative Transcriptomics of Rat and Axolotl After Spinal Cord Injury Dissects Differences and Similarities in Inflammatory and Matrix Remodeling Gene Expression Patterns

**DOI:** 10.3389/fnins.2018.00808

**Published:** 2018-11-13

**Authors:** Jure Tica, Athanasios Didangelos

**Affiliations:** ^1^Sir Alexander Fleming Building, Imperial College London, London, United Kingdom; ^2^Department of Infection, Immunity & Inflammation, University of Leicester, Leicester, United Kingdom

**Keywords:** axolotl regeneration, spinal cord injury, tissue injury, systems biology, network analysis

## Abstract

Following spinal cord injury in mammals, maladaptive inflammation, and matrix deposition drive tissue scarring and permanent loss of function. In contrast, axolotls regenerate their spinal cord after severe injury fully and without scarring. To explore previously unappreciated molecules and pathways that drive tissue responses after spinal cord injury, we performed a 4-way intersection of rat and axolotl transcriptomics datasets and isolated shared genes with similar or differential expression at days 1, 3, and 7 after spinal cord injury in both species. Systems-wide differences and similarities between the two species are described in detail using public-domain computational tools and key differentially regulated genes are highlighted. Amongst persistent differential expression in matching neuronal genes (upregulated in axolotls but downregulated in rats) and nucleic acid metabolism genes (downregulated in axolotls but upregulated in rats), we found multiple extracellular matrix genes that were upregulated in both species after spinal cord injury and all time-points (days 1, 3, and 7), indicating the importance of extracellular matrix remodeling in wound healing. Moreover, the archetypal transcription factor SP1, which was consistently upregulated in rats but was unchanged in axolotls, was predicted as a potential transcriptional regulator of classic inflammatory response genes in rats most of which were not regulated in regenerating axolotls. This analysis offers an extensive comparative platform between a non-regenerating mammal and a regenerating urodele after spinal cord injury. To better understand regeneration vs. scarring mechanisms it is important to understand consistent molecular differences as well as similarities after experimental spinal cord injury.

## Introduction

Following spinal cord injury (SCI) in mammals, inflammation and reactive gliosis drive neuronal loss and irreversible tissue scarring (Fitch and Silver, 2008). The maladaptive inflammatory response and accumulation of extracellular matrix proteins in mammalian lesions are largely responsible for the lack of neuronal regeneration after SCI (Gaudet and Popovich, 2014). Tissue remodeling mechanisms are not well understood and there are no therapies that promote functional repair in mammals. In contrast, urodela such as axolotls and newts have the ability to fully regenerate most tissues including the spinal cord (Echeverri and Tanaka, 2002; Diaz Quiroz et al., 2014; Rodrigo Albors et al., 2015; Sabin et al., 2015). While the mechanisms behind this ability are not fully understood, older and recent work point toward the molecular effect of progenitors, excellent spatiotemporal patterning and matrix remodeling, and an effective immune response (Echeverri and Tanaka, 2002; Monaghan et al., 2007; Godwin et al., 2013). Mammalian SCI has not been compared extensively to axolotls, yet such comparisons have shown the potential to identify molecules and mechanisms with pathological or regenerative function in SCI (Monaghan et al., 2007; Diaz Quiroz et al., 2014).

## Methods

### Rat and axolotl microarray data

Curated rat and axolotl SCI datasets are available online via GEO-NCBI. Rat T7 spinal clip-impact compression injury microarray, 4 sham vs. 4 injured spinal cord samples at 1, 3, and 7 days post-injury (https://www.ncbi.nlm.nih.gov/geo/query/acc.cgi?acc=GSE45006). Microarray gene expression profiling of axolotl SCI (3 uninjured vs. 3 injured spinal cord replicates; each replicate is a pool of 10 axolotl spinal cords) at 1, 3, and 7 days post-injury (https://www.ncbi.nlm.nih.gov/geo/query/acc.cgi?acc=GSE71934). Axolotl microarray IDs were matched to human orthologous genes as detailed in Huggins et al. (2012) and Monaghan et al. (2007). These were then used to identify rat orthologs from the rat microarray. T-test *p*-values and fold-changes for rats and axolotls and each time-point are summarized in Supplemental Tables 1–6. Only orthologous genes with adjusted (Benjamini-Hochberg false discovery correction) *p-*value ≤ 0.05 and fold-change cut-off ≥1.5(±) were included.

### Computational analysis of transcriptomics data

Protein-protein interaction networks were created using StringDB-v10.5 (Szklarczyk et al., 2015) from known and predicted protein-protein interactions, using broad threshold of association (0.15–0.999) to capture most possible protein interactions. We used human as species background to maintain analytical consistency, given that the axolotl gene microarray platform has been matched to human orthologs. Moreover, human functional annotations offer generally superior coverage and research data available. Network parameters were visualized in CytoScape-v3.6 (Smoot et al., 2011). Full gene ontology (GO) enrichment analysis, which combines “biological process,” “molecular function,” and “cellular component” categories, was derived using BinGO (Maere et al., 2005) in CytoScape. Homo Sapiens was also used as a reference list for GO enrichment. Transcription factor analysis was performed using MSigDB transcription factor targets (TFT) sub-collection (Liberzon et al., 2015). Graphs and statistics were completed using GraphPad Prism-v7 and MatLab-R2017b. SPIA-v2.32.0 (Tarca et al., 2009), was performed in Bioconductor (v3.7; R-v3.5.1).

## Results

To identify molecular differences and similarities between rats and axolotls we performed an intersection of differentially regulated genes identified in rat clip-impact compression SCI microarray performed by Chamankhah et al. (2013) at 3 time-points after injury (days 1, 3, and 7) with orthologous differentially regulated genes identified in a microarray dataset of axolotl SCI (spinal cord ablation) performed by Sabin et al. (2015) also at 1, 3, and 7 days post-injury. Microarray data is listed in Supplemental Tables 1–6. Detailed quantitative cross-species and temporal comparison is summarized in Supplemental Figures 1A,B. Overall, rats and axolotls are very different to each other while individual post-injury time-points within species are quite similar. Most of the data variance is generated due to species rather than temporal differences (Supplemental Figures 1C–H).

**Figure 1:**
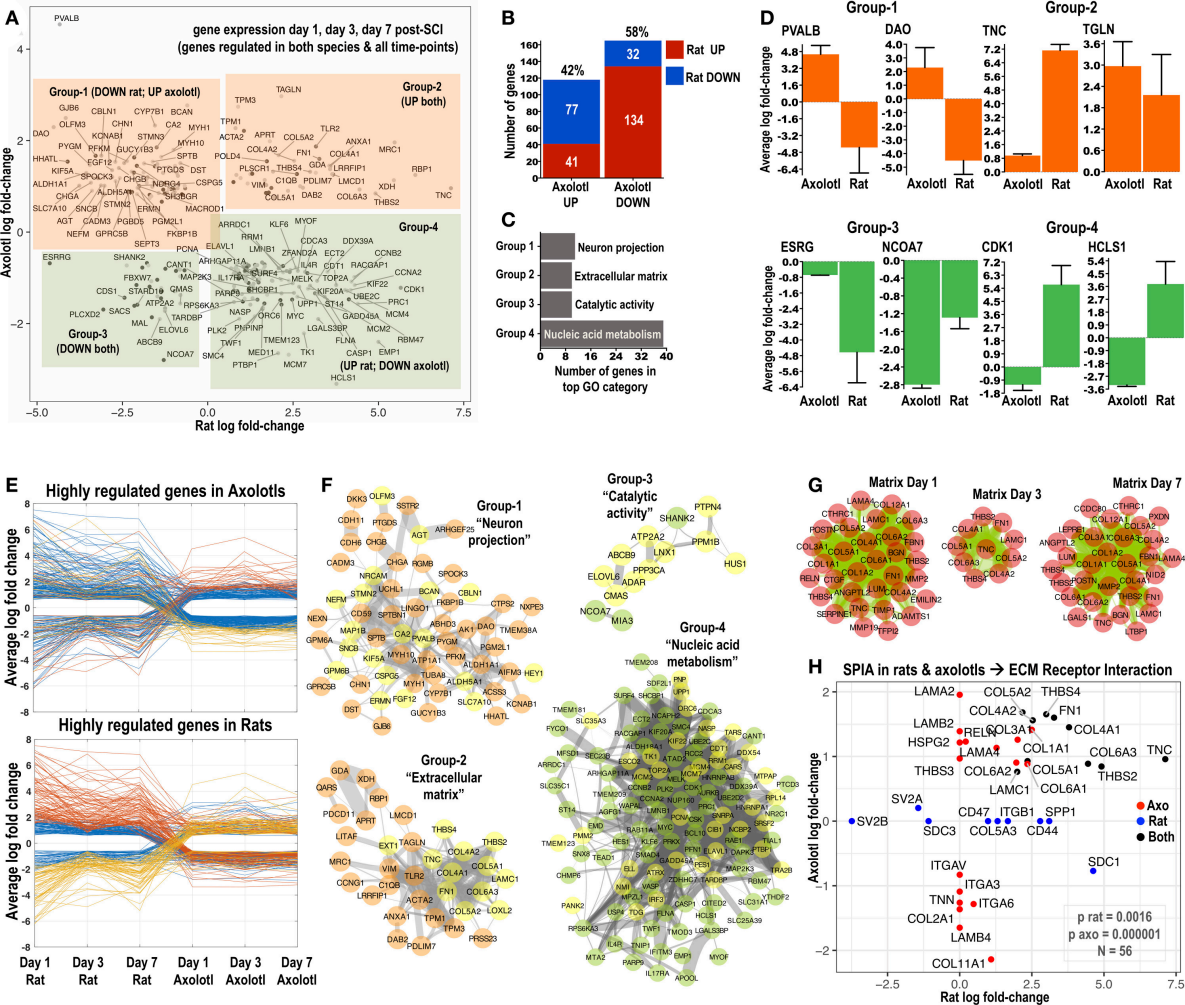
Identification of common differentially regulated genes in rats and axolotls after SCI. **(A)** The 4-direction scatter-plot depicts shared differentially regulated genes identified in the rat and axolotl microarray datasets at 1, 3, and 7 days post-SCI. Only significantly altered transcripts were included (all expression data in Supplemental Tables 1–6). Shared differentially regulated transcripts were split into 4 groups according to their log fold-change in rat and axolotl microarrays: Group-1 genes were upregulated in axolotls but downregulated in rats; Group-2 genes were upregulated in both species; Group-3 genes were downregulated in both species and Group-4 genes were upregulated in rats but downregulated in axolotls. Highly differentially regulated genes are labeled. **(B)** Number and percentage of shared genes from the intersection of rat and axolotl microarrays 1, 3, and 7 days post-SCI and their distribution between the species. **(C)** The chart summarizes the most overrepresented GO term for Groups 1–4 shown in **(A)**. Full GO enrichment analysis (combining “biological process,” “molecular function,” and “cellular component” categories) was performed with BinGO in Cytoscape. All overrepresented GO terms are summarized in Supplemental Figure 2. Homo Sapiens was used as the reference organism for GO enrichment. **(D)** The most highly differentially regulated genes from Groups 1–4 and for each species are shown. y-axis is the average log-fold change for each gene for days 1, 3, and 7. Note that for these top regulated genes, gene expression changes in rats are somehow more pronounced than axolotls. **(E)** Parallel coordinate plots visualize differentially regulated genes in rats and axolotls (cut-off −1.5 and +1.5 log fold-change) and how they change across the species from day 1 to day 7. **(F)** Protein-protein interaction networks isolating genes that comprise Groups 1–4 from **(A)**. Networks were made using StringDB (Homo Sapiens as the background organism) and visualized in Cytoscape using organic biolayout. Only connected nodes were included and those that did not connect were left out. Thickness of edges is protein-protein interaction probability derived from StringDB (0.15; thinnest to 0.999; widest). Note that low probabilities (0.15–0.40; thin lines) might represent low confidence protein associations not based on physical protein-protein interactions but likely derived from less reliable text-mining data. Yellow nodes highlight genes that belong to the top GO category for each group, which is also indicated. **(G)** Protein-protein interaction networks depict shared upregulated extracellular matrix transcripts at day 1, day 3, and day 7 post-SCI in rats and axolotls. Note that while 35 matrix genes are upregulated in both species at day 1 and day 7, only 10 are upregulated in both species at day 3 post-SCI (see also Supplemental Figures 1I-K). **(H)** Signaling pathway impact analysis (SPIA) of consistently differentially regulated genes (days 1, 3, and 7) in rats and axolotls. “ECM Receptor Interaction” was significant in both species post-SCI. The axolotl appears to have a smaller *p*-value for this pathway given that more matrix proteins are consistently (days 1, 3, and 7) differentially regulated in the regenerating species. The scatter plot displays the 56 genes involved in this pathway and how their average (days 1, 3, and 7) fold-change behaves in the two species (x-axis rat; y-axis axolotl). Red genes are consistently differentially regulated in axolotls but not in rats. Blue are consistently differentially regulated in rats but not in axolotls. Black are regulated consistently in both species. Further SPIA analysis and technical details can be found in Supplemental Figure 3.

To narrow down the list of interesting targets, we isolated genes that were either up or downregulated in both species consistently during all post-injury time-points (days 1, 3, and 7). Thus, we filtered genes with stable differential regulation over time (depicted in Figure 1A). This selection approach has certain advantages: First, it increases the statistical stringency of gene selection by accepting genes only if they are significantly regulated simultaneously in 6 datasets (3 time-points and 2 species). Selection stringency is particularly relevant in this comparison, which involves analysis of datasets derived from 2 highly different species and different labs. Second, consistently regulated shared genes isolate temporally stable molecular patterns that are not drastically changing during the first week post-SCI and are therefore likely important in species' post-SCI responses.

The intersection of differentially regulated genes across time-points and species returned 4 groups of shared genes (Figure 1A): Group-1 with 77 genes upregulated after axolotl SCI but downregulated after rat SCI at all 3 time-points; Group-2 with 41 genes upregulated in both species post-SCI and all 3 time-points; Group-3 with 32 genes persistently downregulated in both species and Group-4 with 132 genes downregulated in axolotls but upregulated in rats. In total, 58% shared genes are downregulated in axolotls and from those the majority (~80%) are actually upregulated in rats (Figure 1B). Similarly, 42% shared genes are upregulated in axolotls and the majority (~64%) are downregulated in rats (Figure 1B) indicating clear cross-species differences after SCI. The most highly regulated shared genes across all time-points for each species are depicted in Figure 1D. Excluding TNC and CDK1 whose upregulation and function after mammalian SCI has been studied in detail (Tian et al., 2006; Lu et al., 2007), the function of PVALB, DAO, TGLN, ESRG, NCOA7, and HCLS1 after SCI is unknown. Multiple highly regulated shared genes follow a reverse expression trend in rats and axolotls (Figure 1E).

Next, full GO enrichment, which combines “biological process,” “molecular function,” and “cellular component” categories, was used to identify overrepresented GO terms in each group of intersected shared genes. Top GO terms are highlighted in Figure 1C. Shared genes from Groups 1 to 4 are visualized as protein-protein interaction networks in Figure 1F. The predominant GO category in Group-1 was “neuron projection” (Figure 1F; neuronal genes highlighted as yellow nodes) indicating spinal cord regeneration in axolotls but inability of rats to regenerate. In Group-2 the top GO is “extracellular matrix” (Figure 1F; yellow highlights matrix genes). Group-3 has a predominant GO “catalytic activity” (Figure 1F) and Group-4 main GO is “nucleic acid metabolism” populated by nuclear genes including multiple transcriptional regulators with diverse roles in gene expression and cell-cycle control. All GO terms from Groups 1 to 4 are visualized in Supplemental Figure 2.

Given that axolotls are able to repair after injury without scarring, gene expression is likely associated with regeneration. Thus, upregulation of neuronal entities in axolotls and concomitant downregulation of these genes in rats (Group-1) is not surprising. Our attention was captured by genes in Group-2 (Figures 1A,F; upregulated in both species). This group contains classic extracellular entities TNC (tenascin-C), FN1 (fibronectin), THBS2/THBS4 (thrombospondins), and LAMC1 (laminin-C1), as well as collagen-4 (COL4A1/A2), collagen-5 (COL5A1/A2), collagen-6 (COL6A3), lysyl-oxidase-2 (LOXL2; collagen biosynthesis) and exostosin-1 (EXT1; heparan-sulfate biosynthesis). There are also few inflammation-related genes including TLR2, MRC1 (CD206), ANXA1 (annexin-A1), C1QB (complement-Q1B) and VIM (vimentin; mesenchymal marker). Notably, at day 1 and 7 there are many more matrix genes expressed in both species (Figure 1G) and their apparent lack of regulation at day 3 in rats, shrinks the shared extracellular cluster (Figure 1G). At days 1 and 7, matrix is enriched with fibrosis-related collagen-1 (COL1A1/A2) and collagen-3 (COL3A1), collagen-associated proteoglycans LUM (lumican), NID2 (nidogen-2), POSTN (periostin), BGN (biglycan), LGALS1 (galectin-1), and COL12A1 (collagen-12; Figure 1G). Overall, this signature indicates active matrix remodeling in both species post-SCI and confirms molecules found in previous studies (Monaghan et al., 2007; Didangelos et al., 2016). Interestingly, when we performed *SPIA* (Tarca et al., 2009) as an alternative approach to identify molecular pathways after SCI (Supplemental Figure 3), “ECM receptor-interaction” was the only common pathway in both species (Figure 1H), containing most intersected upregulated matrix genes from Group-2 (Figure 1F) plus others with differential species expression.

Correlation of the average fold-change of shared upregulated and downregulated rat and axolotl genes (Figure 1A) against measures of interaction network centrality (Supplemental Figure 4) including betweenness-centrality, degree-centrality and clustering-coefficient (as a proxy to the biological power of proteins within a network; Ercsey-Ravasz and Toroczkai, 2010) indicated that the fold-change of genes upregulated in axolotls had a positive and significant correlation with betweenness-centrality and degree-centrality (Supplemental Figures 4A–C). Proteins upregulated in rats were positively and significantly correlated with degree-centrality and clustering-coefficient (Supplemental Figures 4G–I). No correlations were observed with downregulated proteins in axolotls or rats (Supplemental Figures 4D–F,J–L). It is likely that genes with central biological function (predicted from network topography) tend to be overexpressed after injury. Few highly central genes (CA2, PVALB, MYC, TOP2A, CDK1) were consistently expressed (day 1, 3, and 7) in the opposite direction in rats and axolotls, while other central genes (TLR2, ACTA2, FN1, TNC, VIM) were consistently upregulated in both species, highlighting the complexity of molecular mechanisms post-SCI.

**Figure 2:**
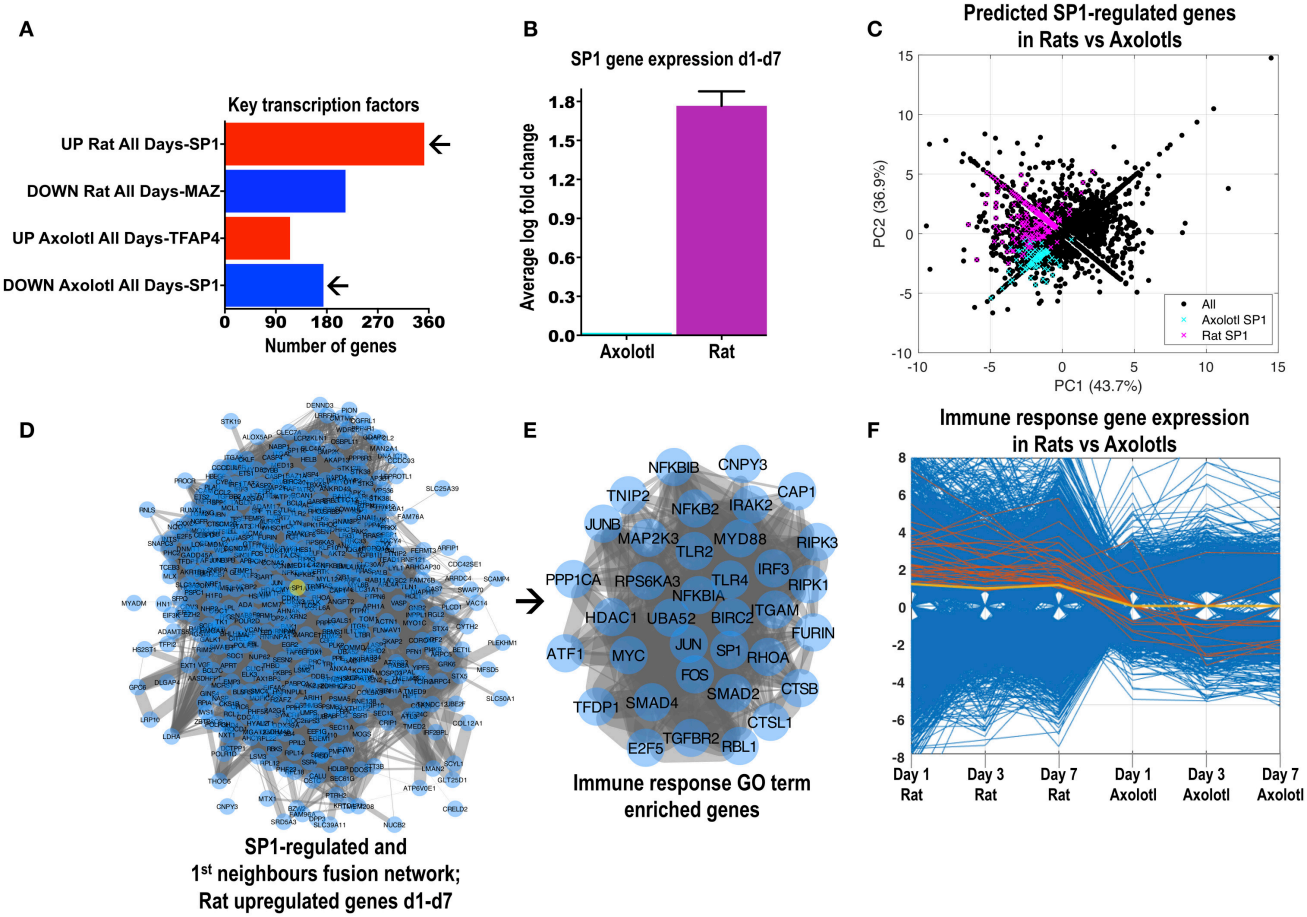
The transcription factor SP1 likely controls a large number of differentially regulated genes in rats and axolotls. **(A)** Transcription factors with the highest number of likely promoter binding sites. Transcription factor mining was performed with MSigDB. This tool counts genes having one or more occurrences of transcription factor binding sites in regions spanning 4 kb around their transcription starting sites. SP1 was the transcription factor with the highest number of likely hits and lowest adjusted *p*-value for genes that were consistently upregulated in rats but consistently downregulated in axolotls at days 1, 3, and 7 post-SCI. **(B)** SP1 gene expression was increased in rats after SCI but was unchanged in axolotls at days 1, 3, and 7 post-SCI. The graph depicts the average (days 1, 3, and 7) log fold-change in the expression of SP1 from the rat and axolotl microarray datasets. Rat and axolotl SP1 sequences share a 65% identity and 78% similarity. **(C)** Principal component analysis of all differentially regulated genes in rats and axolotls highlighting the genes that were predicted by MSigDB to contain SP1 promoter binding sites. Magenta highlights rat SP1-controlled genes while blue highlights axolotl SP1-controlled genes. **(D)** Protein-protein interaction network of 316 SP1-regulated genes and 284 SP1 1st neighbor genes (see Supplemental Figures 5A–C). All 514 genes are upregulated after rat SCI at day 1, 3, and 7. SP1 is highlighted as a yellow node. **(E)** The SP1-related genes shown in **(D)** contained a tightly connected cluster of classic inflammatory response genes (“immune system process;” see Supplemental Figure 5D) visualized as a protein-protein interaction network. Note the presence of classic inflammatory response genes, signaling components and transcription factors. **(F)** The parallel coordinate plot visualizes the inflammatory response genes shown in **(E)** in rats and axolotls and how they change across the species from day 1 to day 7 (orange lines). Most of these genes are upregulated in rats but downregulated or unchanged in axolotls. SP1 is the yellow line.

One important question is how the similar or different cross-species responses are modulated after SCI. In an attempt to dissect this further we analyzed differentially regulated genes for transcription factor binding sites (MSigDB). The archetypal transcription factor SP1, was predicted to bind promoter sites for the greatest number of genes upregulated in rats (316; Figure 2A) but also the greatest number of genes downregulated in axolotls (162). Interestingly, SP1 was upregulated in rats at all 3 time-points but was not regulated in axolotls (Figure 2B). Shared rat and axolotl genes with likely SP1-binding sites are generally upregulated in rats (Figure 2C). To further investigate *in silico* a potential role for SP1 in rats, we combined upregulated genes MSigDB-predicted to have SP1-binding sites (Supplemental Figure 5A) with SP1 first-neighbors (Supplemental Figure 5B) derived from the network of upregulated rat genes. The fused SP1 network (514 genes) is shown in Figure 2D. The SP1 interactome was subjected to GO enrichment (Supplemental Figures 5C–E) where we noted a group of genes typically involved in inflammation (extracted in Figure 2E). This cluster included pattern-recognition receptors TLR2 and TLR4 (studied in SCI; Gensel et al., 2015), adaptors IRAK2, MYD88, and BIRC2, NF-kappaB components NFKB2, NFKBIA, NFKBIB, TNIP2, RIPK1, and RIPK3, inflammatory regulation transcription factors JUN, JUNB, FOS and IRF3, as well as TGFB signaling elements, TGFBR2, SMAD2, SMAD4, SP1, and even MYC (Di Giovanni et al., 2003). Importantly, excluding TLR2 and few others, these genes are either downregulated or not regulated in the axolotl after SCI (Figure 2F). Thus after rat SCI, SP1 might control classic inflammatory signaling genes. SPIA analysis also identified “TLR signaling” as a significant activated pathway in rats after SCI with multiple overlapping genes (Supplemental Figure 3A), corroborating the SP1-related inflammatory signature (Figure 2E). Importantly, axolotl SP1 (derived from 4 contigs found in www.ambystoma.org matched to human refseq) has a 65% identity and 78% similarity with rat SP1 suggesting a comparable function in both species (rat and human SP1 are 96% identical). Nevertheless, the possible transcriptional role of SP1 in axolotls is currently unknown and might be different to mammals.

## Discussion

There are two observations that we found particularly interesting and in our opinion warrant further examination. First, the consistent upregulation of multiple matrix remodeling genes in both species after SCI, many of which have unknown function. The importance of matrix gene expression has been noted previously in a study comparing axolotl tail amputation with rat contusive SCI at different time-points (Monaghan et al., 2007). Matrix accumulation is perhaps counterintuitive in terms of axolotl regeneration given the negative function of fibrosis in mammalian wounds. Matrix remodeling might be a species-conserved tissue repair mechanism that drives regeneration in axolotls but is utilized ineffectively and leads to scarring in mammals. In contrast to mammals, fibrosis in axolotls is transient and remodeling of early scars by macrophages and fibroblasts leads to complete tissue restoration (Godwin and Rosenthal, 2014; McCusker et al., 2015).

Second, SP1, which was consistently upregulated after rat SCI but was not regulated in axolotls, might be an important regulatory factor in SCI. Although SP1 controls a plethora of genes in mammals, including fibrotic and inflammatory entities (Verrecchia et al., 2001; Kaczynski et al., 2003), its function after SCI is not well-characterized and needs to be investigated further. The relationship of SP1 with classic inflammatory genes upregulated in rats suggests that it might participate in the maladaptive inflammatory response that affects the mammalian system after SCI leading to neuronal cytotoxicity and scarring. In contrast, urodela exhibit a highly effective and regulated immune response, which is non-cytotoxic and resolves quickly following tissue injury. This is perhaps related to the fact that axolotls lack adaptive immune capacity and might explain why their immune system favors scarless tissue repair and promotes positive matrix remodeling (Godwin et al., 2013, 2017).

More comparative studies are needed to understand the molecular mechanisms that control scarless repair and regeneration in axolotls and to identify molecular factors that differently regulate inflammation and matrix remodeling in urodela vs. mammals. Such studies need to be carefully designed in terms of injury models, which could drastically affect gene expression. Future comparative efforts will be boosted by recent work that sequenced the entire axolotl genome (Nowoshilow et al., 2018) and highlighted the importance of developmental factors including PAX3/7, the HOXA gene cluster, LY6 gene family and others, as key species-restricted determinants of tissue restoration in axolotl limb regeneration. Species-restricted regulators of regeneration should be examined in SCI and studied in the context of the maladaptive mammalian response to understand how such factors might be modulated in mammals to enhance functional repair.

In summary, we performed a 4-way comparison of consistent gene expression in rats and axolotls after SCI and identified previously unexplored molecules and pathways that might define successful or failed regeneration.

## Author contributions

AD conceived and designed the study, performed data analysis, and wrote the manuscript. JT performed data analysis and wrote parts of the manuscript.

### Conflict of interest statement

The authors declare that the research was conducted in the absence of any commercial or financial relationships that could be construed as a potential conflict of interest.
