# Supplementary material for: Comparative Transcriptomics of Rat and Axolotl After Spinal Cord Injury Dissects Differences and Similarities in Inflammatory and Matrix Remodeling Gene Expression Patterns
Source: Front Neurosci. 2018 Nov 13;12:808. doi: 10.3389/fnins.2018.00808 (PMC6262295; doi:10.3389/fnins.2018.00808)
Supplement: Supplementary file 7 [file Data_Sheet_1.PDF]

Supplemental Fig. 1

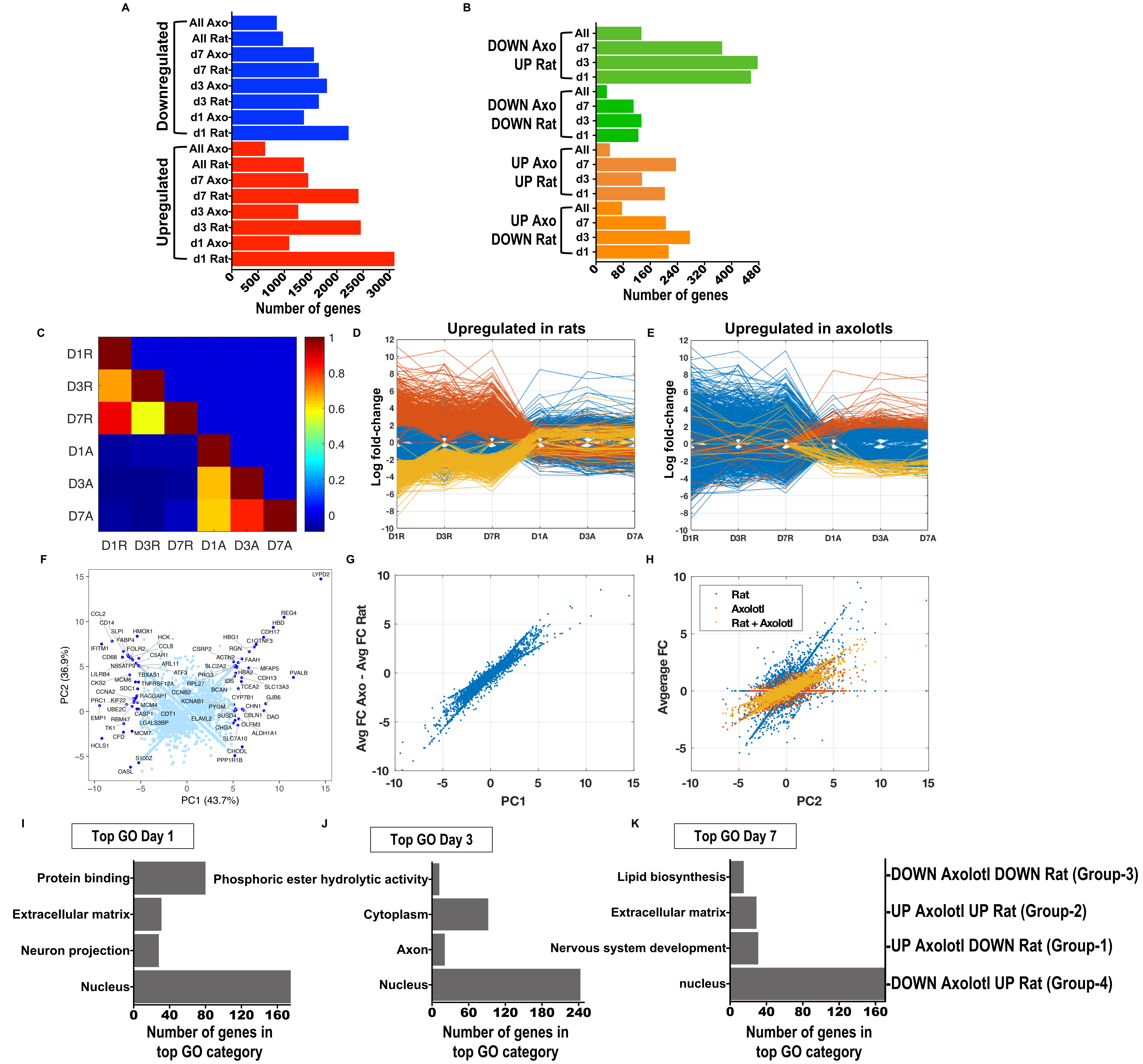

Rat and Axolotl microarray datasets comparison 1, 3 and 7 days after spinal cord injury.

A: Total number of significantly differentially regulated genes (adjusted *p*≤0.05 and fold change -1.5 to 1.5) from all SCI microarray datasets; day 1, day 3 and day 7; rat and axolotl. All expression data in **Supplemental Tables 1-6.** Importantly, note the difference in the numbers of differentially regulated genes between the two species. This is likely the result of the different species microarray platforms and the reduced microarray coverage of axolotl genes in comparison to rats. Thus, cross-species comparisons focusing on intersected genes that are expressed in both species in tandem might be narrowed down by the different number of genes identified by microarray in each species.

B: Total number of differentially regulated orthologous genes from all SCI microarray datasets that are shared between the 2 species at day 1, day 3 and day 7.

C: Covariance matrix of all rat and axolotl significantly differentially regulated genes after spinal cord injury. Gene expression changes correlate well for each species across time-points but poorly between the two species. Day 3 in the rat (D3R) appears to have lower overall change in gene expression.

D-E: Parallel coordinate plots of significantly regulated genes (adjusted *p*≤0.05) with an average fold-change greater than 3 (red) and lower than -3 (yellow) in rats (**D**) and axolotls (**E**) across the 3 time-points. Note that multiple regulated genes are differentially expressed in the two species. The rat displays a greater number of highly differentially regulated genes in comparison to the axolotl.

F-H: Principal component analysis of rat and axolotl gene expression datasets. All gene expression data is projected onto principal components 1 and 2 (PC1 and PC2) as indicated (**F**). The most differentially regulated genes between species are highlighted and labeled. PC1 correlates well with the difference between the average fold-change across time-points between axolotls and rats (**G**) while PC2 correlates well with the average fold-change in gene expression across time-points and species (**H**). Thus, the 6-dimensional dataset (2 species and 3 time-points) can be projected into just 2 dimensions accounting for 80% of the data variance. Both PC1 and PC2 describe the variance generated by species differences and the remaining variance is due to the temporal component.

I-K: The number of genes in the top GO annotation category for each comparison between rat and axolotl SCI at Day 1, 3 and 7 separately (see Groups 1-4; **Fig. 1**). We used “Full GO” analysis (includes “biological process, “molecular function” and “cellular component”) using BinGO in cytoscape.
