# Supplementary material for: Comparative Transcriptomics of Rat and Axolotl After Spinal Cord Injury Dissects Differences and Similarities in Inflammatory and Matrix Remodeling Gene Expression Patterns
Source: Front Neurosci. 2018 Nov 13;12:808. doi: 10.3389/fnins.2018.00808 (PMC6262295; doi:10.3389/fnins.2018.00808)
Supplement: Supplementary file 8 [file Data_Sheet_2.PDF]

Supplemental Fig. 2

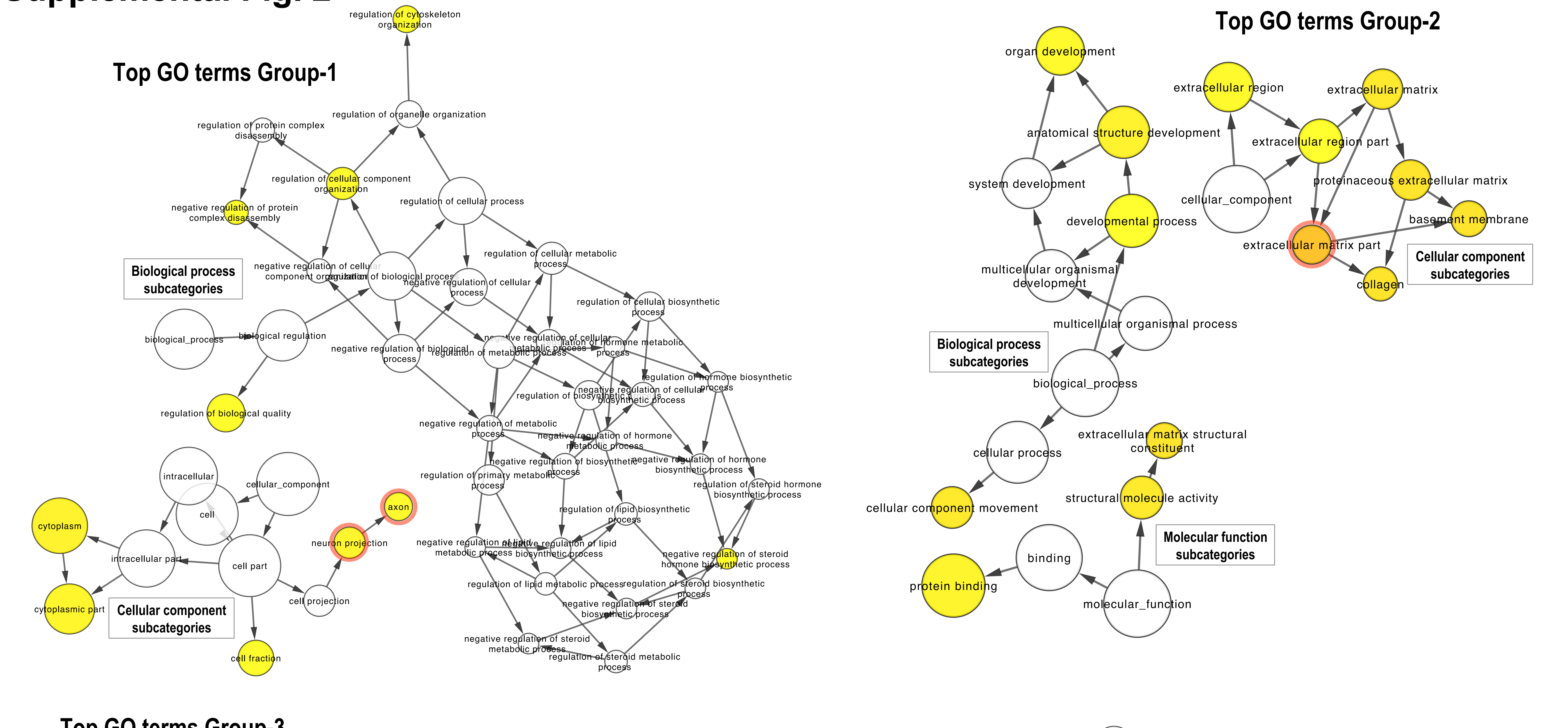

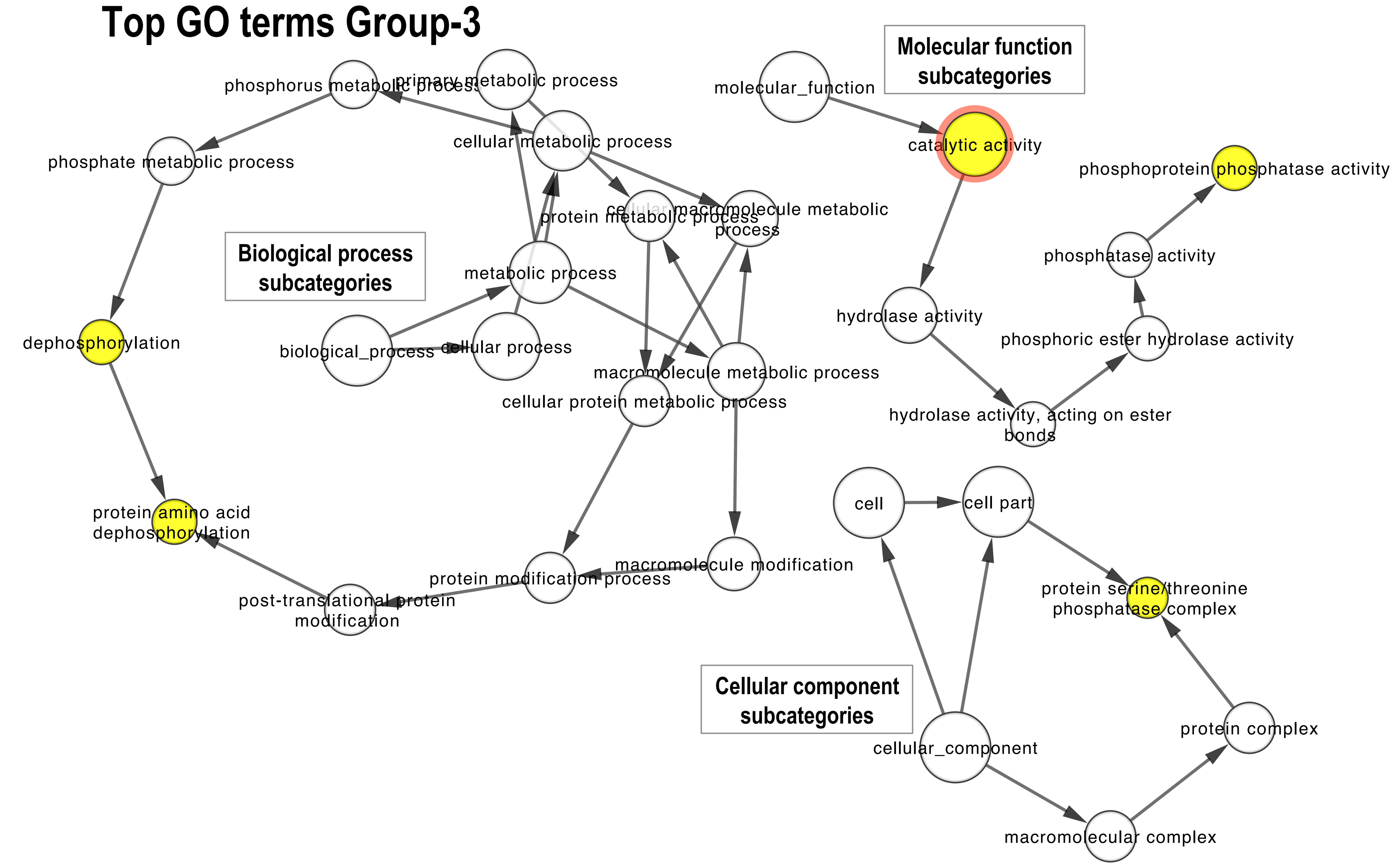

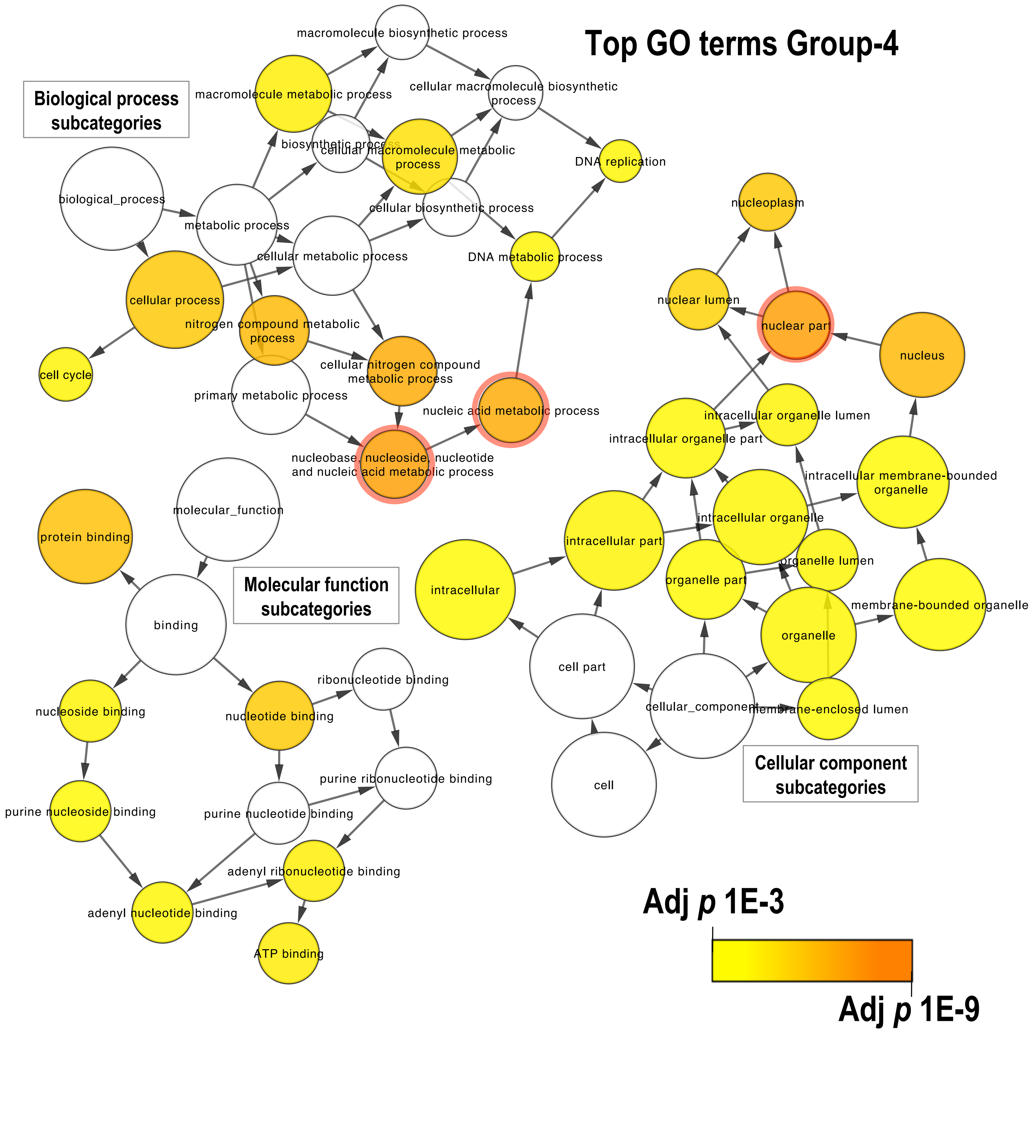

Top gene ontology (GO) annotations for Groups 1-4 of shared differentially regulated genes in rats and axolotls. Top overrepresented GO terms for each Group (Groups 1-4) of shared differentially regulated genes between rats and axolotls for days 1, 3 and 7 after SCI (see **Fig. 1** main manuscript). Full GO analysis was performed using BinGO in Cytoscape. Full GO combines all 3 GO categories "biological process", "molecular function" and "cellular component" as indicated above. GO categories are visualised as feedback networks. The size of nodes represents the number of genes in each GO category and subcategory while the colour is adjusted *p*-value of each overrepresented term as indicated in the key (lower left). Adjusted p-value was computed in BinGO using standard hypergeometric test and Benjamini-Hochberg false discovery rate correction.
