# Supplementary material for: Comparative Transcriptomics of Rat and Axolotl After Spinal Cord Injury Dissects Differences and Similarities in Inflammatory and Matrix Remodeling Gene Expression Patterns
Source: Front Neurosci. 2018 Nov 13;12:808. doi: 10.3389/fnins.2018.00808 (PMC6262295; doi:10.3389/fnins.2018.00808)
Supplement: Supplementary file 9 [file Data_Sheet_3.PDF]

Supplemental Fig. 3

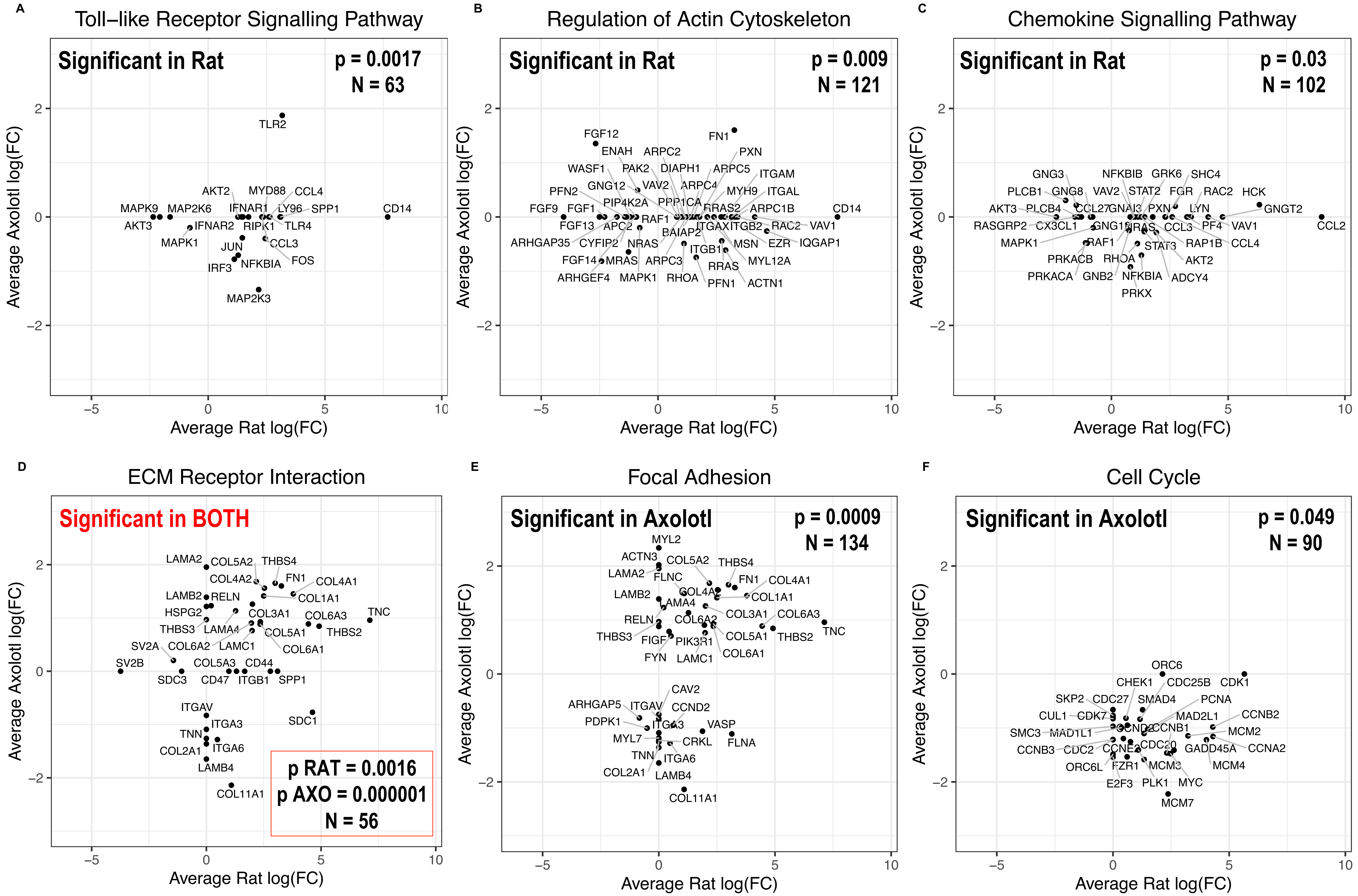

Signalling Pathway Impact analysis (SPIA) examining molecular signatures in gene expression in rats and axolotls. Rat and axolotl microarray analysis genes that were consistently differentially regulated at all 3 time-points (days 1, 3 and 7) were submitted to SPIA. This Bioconductor-based computational tool is using the fold-change of regulated genes to compute likely represented pathways. “TLR Signalling” (**A**) “Regulation of Actin Cytoskeleton” (**B**) and “Chemokine Signalling” (**C**) were significant in rats. “ECM Receptor Interaction” (**D**) was significant in both rats and axolotls while “Focal Adhesion” (**E**) and “Cell Cycle” (**F**) were significant in axolotls. Scatter-plots depict the average log fold-change (days 1, 3 and 7) of differentially regulated genes that make up these pathways and how they behave in rats (gene log fold-change in x-axis) and axolotls (gene log fold-change y-axis). Note that most genes present in **Group-2** (**Fig.1F** ; intersected genes upregulated in rats and axolotls) are part of (**D**) “ECM Receptor Interaction”. Thus, both species converge in the regulation of matrix molecules and the axolotl appears to be more significant given that more matrix genes are upregulated in the regenerating species consistently during days 1, 3 and 7. Most (nuclear) genes present in **Group-4** (**Fig.1F** ; intersected genes upregulated in rats but downregulated in axolotls) are found in (**F**) “Cell Cycle”. Otherwise the rat is enriched in inflammatory entities (**A** and **C**). This is important in the context of **Fig.2** in main manuscript.

SPIA was performed using consistently differentially regulated rat and axolotl genes during days 1, 3 and 7 after SCI. The analysis was conducted on a Homo Sapiens background with a significance threshold of 0.05, nB value of 2000, and combining evidence measures with Fisher’s method. See Tarca et al., 2009 in main manuscript.
