# Supplementary material for: Comparative Transcriptomics of Rat and Axolotl After Spinal Cord Injury Dissects Differences and Similarities in Inflammatory and Matrix Remodeling Gene Expression Patterns
Source: Front Neurosci. 2018 Nov 13;12:808. doi: 10.3389/fnins.2018.00808 (PMC6262295; doi:10.3389/fnins.2018.00808)
Supplement: Supplementary file 10 [file Data_Sheet_4.PDF]

Supplemental Fig. 4

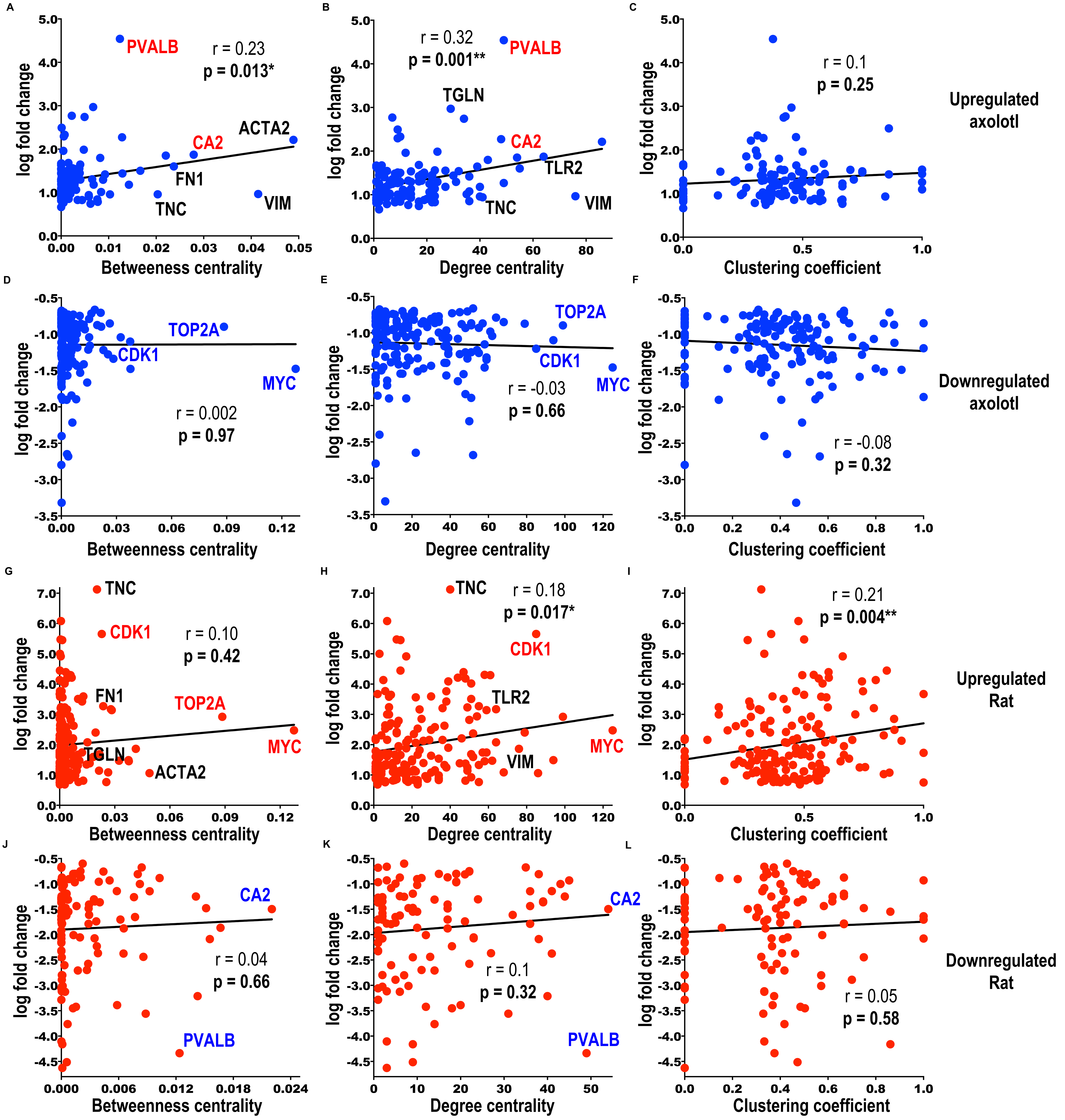

Correlation of gene expression (log fold-change) with measures of network centrality for rat and axolotl differentially regulated genes.
A-F: Pearson correlation plots of the average (days 1, 3 and 7) log fold-change (y-axis) of consistently differentially regulated genes in axolotls after SCI versus their **betweeness centrality** (**A, D**; measures how many times a protein is acting as a shortest path link connecting other proteins in its network neighbourhood. Biologically essential proteins tend have a high betweeness centrality), **degree centrality** (**B, E**; simple number of connections per protein. Similar to betweenness centrality, biologically important proteins are likely to interact with multiple other proteins) and **clustering coefficient** (**C, F;** measures how many connections exist between first neighbours of proteins). **A-C** depict **upregulated** axolotl genes while **D-F** depict **downregulated** axolotl genes.
G-L: As above but plots depict differential regulation of shared intersected genes in rats after SCI. (**G, J**) **betweeness centrality**, (**H, K**) **Degree centrality** and (**I, L**) **clustering coefficient**. **G-I** depict **upregulated** rat genes while **J-L** depict **downregulated** rat genes.
Best fit lines, Pearson *r* and *p* values are indicated. Betweeness centrality, degree centrality and clustering coefficient were calculated in Cytoscape using the protein-protein interaction network of all 284 shared rat and axolotl consistently differentially regulated genes (from days 1, 3 and 7). The network was imported from StringDB v10. Probability threshold was set from 0.15 to 0.999 to cover the widest possible range of interactions and to enhance correlation results. Few shared genes are indicated. Note how some highly central genes (i.e. CA2, MYC, PVALB, TOP2A, CDK1) are expressed in the opposite direction between rats and axolotls during days 1, 3 and 7 post-SCI while others (i.e. FN1, TNC, TLR2, VIM, ACTA2) are expressed in the same direction in both species.
