# Supplementary material for: Comparative Transcriptomics of Rat and Axolotl After Spinal Cord Injury Dissects Differences and Similarities in Inflammatory and Matrix Remodeling Gene Expression Patterns
Source: Front Neurosci. 2018 Nov 13;12:808. doi: 10.3389/fnins.2018.00808 (PMC6262295; doi:10.3389/fnins.2018.00808)
Supplement: Supplementary file 11 [file Data_Sheet_5.PDF]

Supplemental Fig. 5

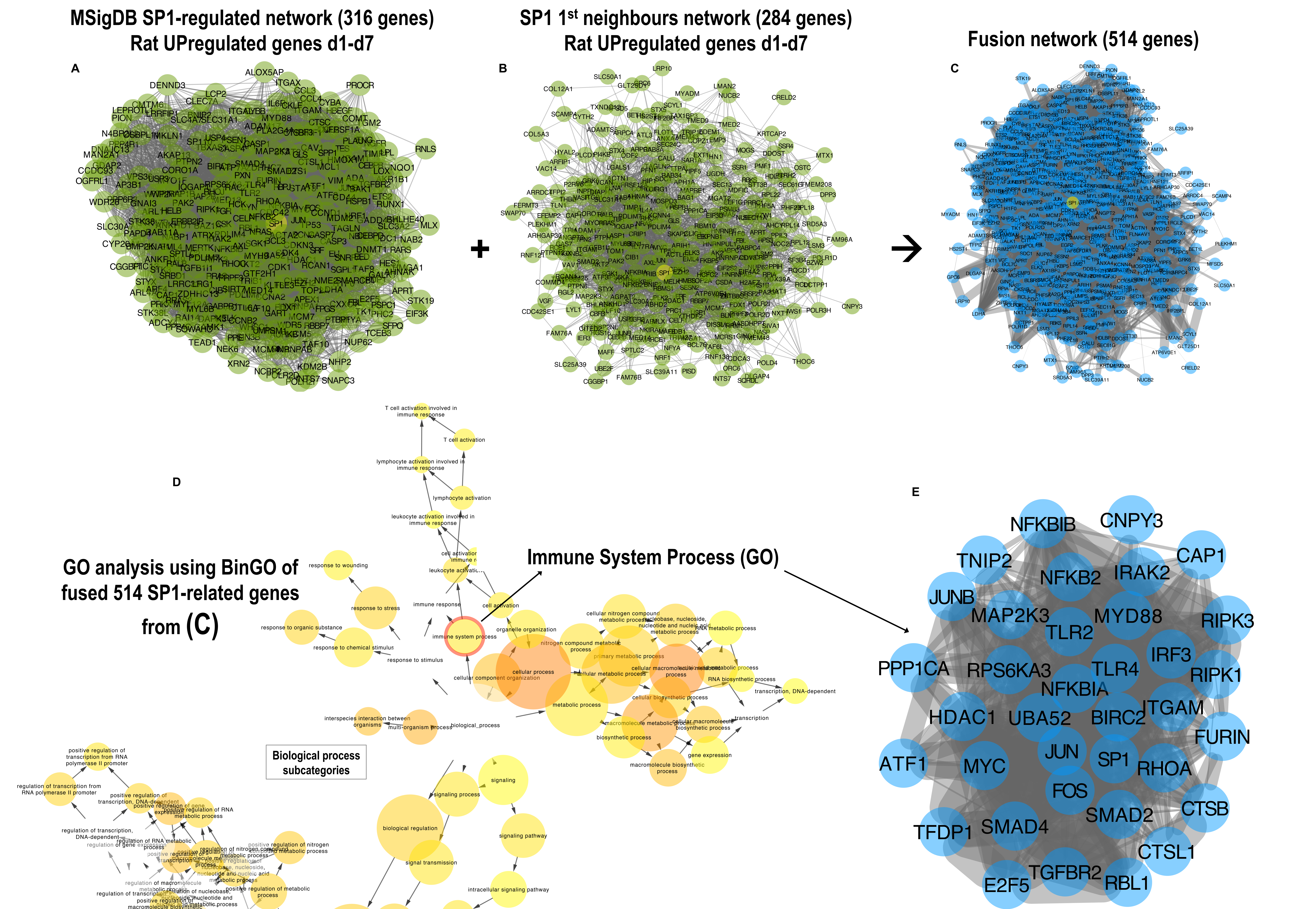

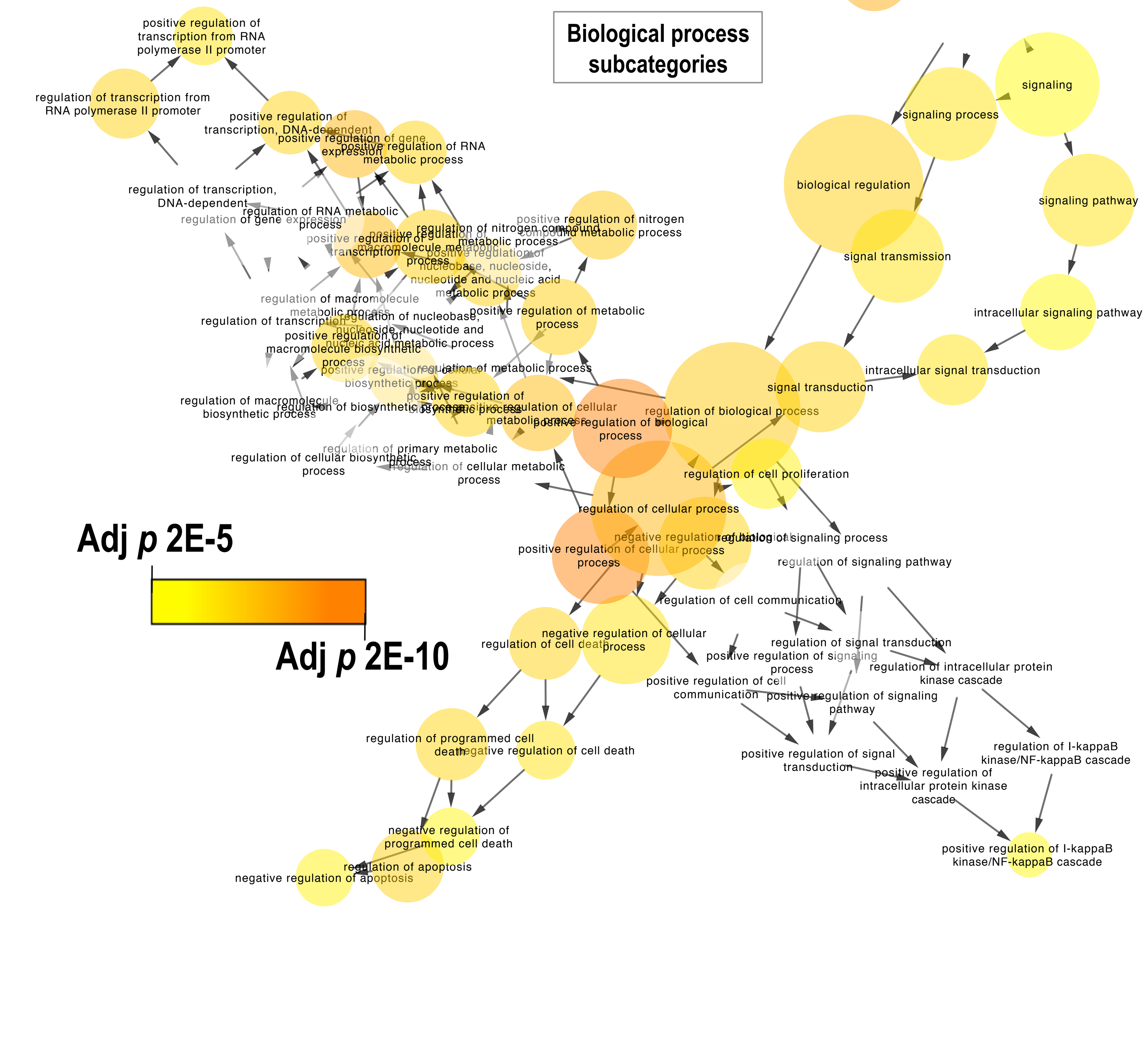

Likely SP1 regulatory component after rat SCI.

A: Protein-protein interaction network of the 316 genes upregulated in rats consistently at days 1, 3 and 7 after SCI and predicted (MSigDB) to contain SP1 promoter binding sites (see **Fig. 2A** main manuscript).

B: Protein-protein interaction network of the 284 genes that are the 1st neighbours of SP1 from the network of consistently (days 1, 3 and 7) upregulated genes in rats after SCI.

C: The fusion of (**A**) and (**B**) results in a network of 514 genes. Interestingly, there is limited overlap between networks in (**A**) and (**B**) with only 82 genes**.**

D: BinGO was used in Cytoscape to examine the most overrepresented GO terms found in the fused SP1 network shown in (**C**). Full GO analysis was performed on (C). "Biological process" GO category is summarised to highlight the "immune system process" subcategory.

E: "Immune system process" GO subcategory is highlighted and the genes that make up this term are gathered in a protein-protein interaction network. Note the presence of classic inflammatory response genes, signalling components and transcription factors.
